# Chinese Herbal Medicine *Ganoderma tsugae* Displays Potential Anti-Cancer Efficacy on Metastatic Prostate Cancer Cells

**DOI:** 10.3390/ijms20184418

**Published:** 2019-09-08

**Authors:** Wen-Chin Huang, Meng-Shiun Chang, Shih-Yin Huang, Ching-Ju Tsai, Pin-Hung Kuo, Han-Wen Chang, Sheng-Teng Huang, Chao-Lin Kuo, Shou-Lun Lee, Ming-Ching Kao

**Affiliations:** 1 Graduate Institute of Biomedical Sciences, School of Medicine, China Medical University, Taichung 40402, Taiwan; 2 Department of Biological Science and Technology, College of Biopharmaceutical and Food Sciences, China Medical University, Taichung 40402, Taiwan; 3 Department of Chinese Medicine, China Medical University Hospital, Taichung 40402, Taiwan; 4 Research Center for Traditional Chinese Medicine, Department of Medical Research, China Medical University, Taichung 40402, Taiwan; 5 Chinese Medicine Research Center, China Medical University, Taichung 40402, Taiwan; 6 Research Center for Chinese Herbal Medicine, China Medical University, Taichung 40402, Taiwan; 7 Department of Chinese Pharmaceutical Sciences and Chinese Medicine Resources, College of Chinese Medicine, China Medical University, Taichung 40402, Taiwan

**Keywords:** anti-cancer efficacy, prostate cancer, metastasis, *Ganoderma tsugae*, Chinese herbal medicine

## Abstract

Resistance to the current therapies is the main clinical challenge in the treatment of lethal metastatic prostate cancer (mPCa). Developing novel therapeutic approaches with effective regimes and minimal side effects for this fatal disease remain a priority in prostate cancer study. In the present study, we demonstrated that a traditional Chinese medicine, quality-assured *Ganoderma tsugae* ethanol extract (GTEE), significantly suppressed cell growth and metastatic capability and caused cell cycle arrest through decreasing expression of cyclins in mPCa cells, PC-3 and DU145 cells. GTEE also induced caspase-dependent apoptosis in mPCa cells. We further showed the potent therapeutic efficacy of GTEE by inhibiting subcutaneous PC-3 tumor growth in a xenograft model. The in vitro and in vivo efficacies on mPCa cells were due to blockade of the PI3K/Akt and MAPK/ERK signaling pathways associated with cancer cell growth, survival and apoptosis. These preclinical data provide the molecular basis for a new potential therapeutic approach toward the treatment of lethal prostate cancer progression.

## 1. Introduction

Prostate cancer (PCa) remains the second leading cause of cancer-related mortality in the Western world [1]. One of the great challenges and clinical issues in PCa study is that the cancer progresses to the deadly metastatic prostate cancer (mPCa). Currently, the first-line systemic treatment for PCa is androgen deprivation therapy (ADT). However, PCa patients who receive ADT drugs usually suffer severe side effects and inevitably develop fatal progression that no longer responds to hormonal therapy [2,3,4]. Furthermore, the Food and Drug Administration (FDA)-approved new drugs for the treatment of aggressive mPCa, such as abiraterone and enzalutamide, have been demonstrated to provide good outcomes in a short timeframe for mPCa patients, although the effectiveness was also very limited clinically [5,6,7]. Therefore, there is an urgent need to seek alternative strategies to treat lethal mPCa progression with superior efficacy and minimal adverse effects.

Traditional Chinese medicine (TCM) is a healing and herbal system of Oriental medicine and has been built on a foundation with a long history. Currently, several TCM herbal products, such as *Ganoderma*, are considered to be safe and have been applied as an alternative therapeutic approach for the successful treatments of chronic and infectious diseases [8]. *Ganoderma* (known as *Lingzhi*) belongs to the prime grade of TCM and has been used to improve human health, promote life span and cure diseases [8,9]. *Ganoderma tsugae* (GT), a restricted species of *Lingzhi* specifically cultivated in Taiwan, has been shown to have high antioxidant activity and treat cardiovascular and allergic diseases [10,11]. In addition to the potential functions in these diseases, our laboratory previously demonstrated that GT exhibited antiproliferative and apoptotic effects on several human cancer cells, including colorectal, epidermoid, lung, breast and ovarian cancers [12,13,14,15]. Moreover, we recently found that the ethanol extract of GT significantly inhibited cell growth of mPCa in vitro [16]. However, the clinical benefits and the underlying molecular mechanisms of GT ethanol extract (GTEE) in lethal mPCa progression remain to be elucidated.

The aim of this study is to determine the potential anti-cancer efficacy of GTEE and reveal the molecular mechanisms of GTEE-mediated suppression of proliferation and aggressive behaviors of mPCa cells. The in vitro and in vivo results showed that GTEE suppressed cell proliferation, migration, invasion and tumorigenicity in mPCa PC-3 and DU145 cells as well as inhibited the growth of xenografted PC-3 tumor in mice. The mechanistic data demonstrated that GTEE inhibited the PI3K/Akt and MAPK/ERK axes, induced cell cycle arrest and activated the caspase-apoptotic pathway in mPCa cells. Taken together, these findings shed light on a potential pharmacological perspective of traditional Chinese medicine-GTEE in the treatment of lethal mPCa.

## 2. Results

### 2.1. GTEE Inhibits Cell Growth and Metastatic Capability of mPCa Cells

**Figure 1:**
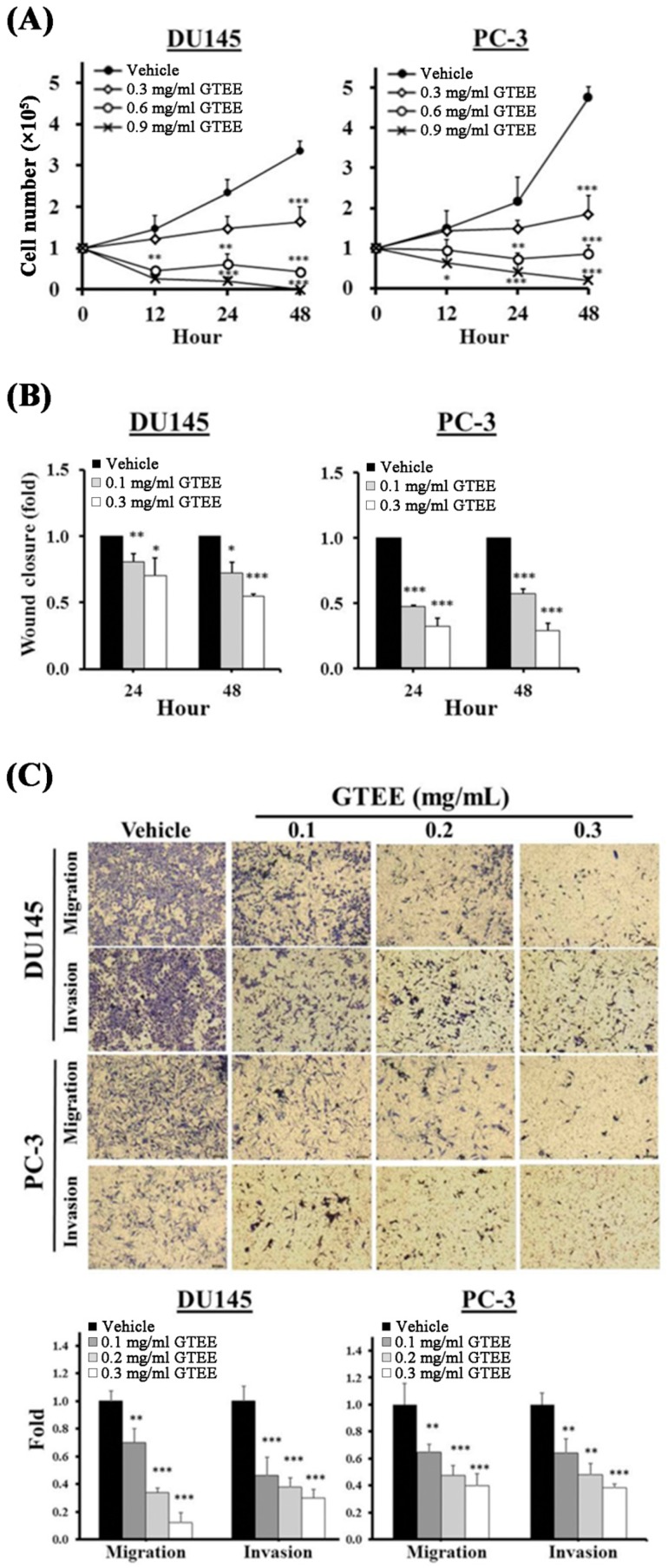
*Ganoderma tsugae* ethanol extract (GTEE) inhibits cell growth and metastatic capability of metastatic prostate cancer (mPCa) cells. (**A**) DU145 and PC-3 cells were treated with vehicle or GTEE (0.3, 0.6 or 0.9 mg/mL) for 0, 12, 24 and 48 h. Cell growth curves were determined by cell number counting. (**B**) A wound-healing assay was used to determine the effect of GTEE on the cell migratory ability. DU145 and PC-3 cells were treated with vehicle or GTEE (0.1 or 0.3 mg/mL). Wound closure was determined by migratory distance at 24 and 48 h. (**C**) The migration and invasion assays of DU145 and PC-3 cells treated with vehicle or GTEE (0.1, 0.2 or 0.3 mg/mL) for 24 h were performed. Images of DU145 and PC-3 cells in migration and invasion transwell assays. Data are shown as the mean ± SD of three independent experiments. * *p* < 0.05, ** *p* < 0.01, *** *p* < 0.001.

To evaluate the anti-mPCa effects of GTEE, we treated mPCa cells, PC-3 [17] and DU145 [18], with various concentrations of GTEE followed by the functional analyses, including cell viability, migration and invasion assays, individually. First, we determined the viability of cells by cell number counting. GTEE significantly decreased the viability of both DU145 and PC-3 cells in a concentration (0.3, 0.6 and 0.9 mg/mL) and a time (0, 12, 24 and 48 h) dependent manner (Figure 1A). The ability to invade surrounding tissues and migrate efficiently is one of the hallmarks of metastatic cancer cells. The principal difference between migration and invasion is that migration refers to cell movement; whereas invasion describes cells actively invading surrounding tissues for metastasis. Subsequently, the effects of GTEE on the migration and invasion potentials of mPCa cells were determined. A wound-healing assay was utilized to evaluate the migratory ability. As shown in Figure 1B, GTEE (0.1 and 0.3 mg/mL) treatment led to a significant inhibition of wound closure in both DU145 and PC-3 cells at 24 and 48 h. Furthermore, the Boyden chamber method was used to determine in vitro migration and invasion. After 24 h treatment, GTEE significantly decreased the migratory and the invasive capabilities of DU145 and PC-3 cells compared to the vehicle group in a dose-dependent pattern (Figure 1C). These data suggest that GTEE is capable of suppressing cell growth and metastatic capability in mPCa cells with no selectivity on both DU145 and PC-3 cells.

### 2.2. GTEE Induces Cell Cycle Arrest and Inhibits Expression of Cell Cycle-Related Proteins in mPCa Cells

**Figure 2:**
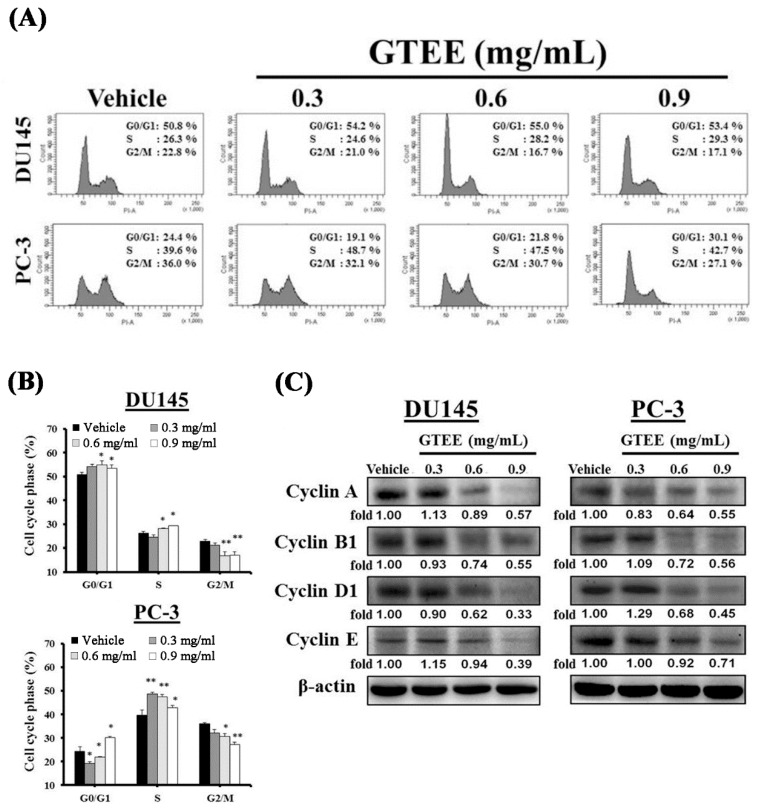
GTEE induces cell cycle arrest and reduces expression of cell cycle-related proteins in mPCa cells. (**A**) DU145 and PC-3 cells were treated with vehicle or GTEE (0.3, 0.6 or 0.9 mg/mL) for 8 h. The cell cycle distribution was measured by flow cytometer assays. (**B**) The quantification of the results from (**A**). Data represent the mean ± SD of three separate experiments. * *p* < 0.05, ** *p* < 0.01. (**C**) DU145 and PC-3 cells were treated with vehicle or GTEE (0.3, 0.6 or 0.9 mg/mL) for 8 h. The total proteins were harvested and expression of cell cycle-related proteins, including cyclin A, B1, D1 and E, were determined by Western blot analysis. β-actin was used as a loading control. The level (fold) of protein expression with the vehicle treatment was assigned as 1.00.

To unravel the mechanism responsible for the inhibition of GTEE-mediated cell growth, the cell cycle distribution was examined by flow cytometric analysis. The results showed that the significant decreases in the numbers of G_2_/M transition cells at concentrations of 0.6 and 0.9 mg/mL GTEE were observed in DU145 cells compared to vehicle-treated cells (Figure 2A,B). In addition, the cell numbers in G_0_/G_1_ transition and S phase were increased by GTEE in DU145 cells (Figure 2B). However, PC-3 cells treated with GTEE resulted in the distinct decreases in the cell numbers of G_0_/G_1_ and G_2_/M transitions compared to vehicle-treated cells (Figure 2B). The decreases in the cell numbers in G_0_/G_1_ and G_2_/M transitions were accompanied by changes in the cell numbers in S phase (Figure 2B). The flow cytometric data indicate that GTEE alters the progression of the cell cycle leading to the inhibition of mPCa cell growth.

Several proteins, such as cyclins, have been demonstrated to regulate cellular pathways associated with the progression of the cell cycle [19]. Next, we assessed the effects of GTEE on the cell cycle-related proteins including cyclins A, B1, D1 and E. Western blotting results showed that GTEE suppressed expression of cyclins A, B1, D1 and E in DU145 cells with a dose-dependent pattern, especially at concentrations of 0.6 and 0.9 mg/mL GTEE (Figure 2C). GTEE also decreased expression of cyclins A, B1, D1 and E in PC-3 cells with a dose-dependent inhibition (Figure 2C). Collectively, these results suggest that the molecular basis of GTEE inhibiting cell growth in mPCa cells is through inducing cell cycle arrest and decreasing expression of various cell cycle protein regulators.

### 2.3. GTEE Inhibits the PI3K/Akt and MAPK/ERK Signaling Pathways Associated with Cell Growth of mPCa

**Figure 3:**
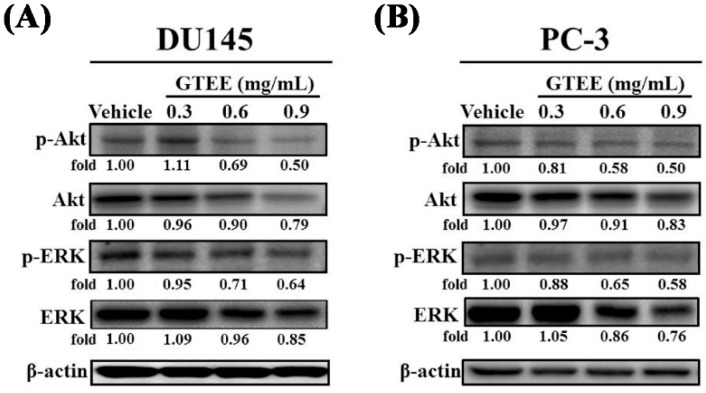
GTEE blocks the PI3K/Akt and MAPK/ERK signaling pathways in mPCa cells. (**A**) DU145 cells and (**B**) PC-3 cells were treated with vehicle or various concentrations (0.3, 0.6 and 0.9 mg/mL) of GTEE for 24 h. The total proteins were prepared from each sample and expression of p-Akt (Ser473), Akt, p-ERK (Thr202/Tyr204) and ERK was determined by Western blot analysis (left panel). β-actin was used as a loading control. The expressed protein levels (p-Akt and p-ERK) were quantified by the ImageJ software (right panel). The level (fold) of protein expression with the vehicle treatment was assigned as 1.00. The results represent the mean ± SD of three independent experiments.

The PI3K/Akt signaling pathway has been demonstrated to be linked to cell growth, the cell cycle and survival in PCa [20,21]. Therefore, we next analyzed the influence of GTEE on the PI3K/Akt signaling pathway in mPCa cells. As shown in Figure 3A,B, GTEE exhibited a dose-dependent inhibitory effect on the levels of phospho-Akt (p-Akt, Ser473) in both DU145 and PC-3 cells. Notably, phosphorylation of Akt (Ser473) has been shown to be associated with cancer aggressiveness and poor clinical outcome in PCa [21,22]. We also observed that GTEE slightly decreased Akt protein at concentration of 0.9 mg/mL in both cell lines after 24 h treatment. Furthermore, the MAPK/ERK signaling pathway has been reported to play an important role in PCa cell proliferation, survival and apoptosis [23,24]. We subsequently examined whether GTEE interrupted the MAPK/ERK signaling pathway. As we expected, GTEE inhibited expression of phospho-ERK (p-ERK, Thr202/Tyr204) in DU145 (Figure 3A) and PC-3 (Figure 3B) cells in a dose-dependent pattern. ERK protein expression was also reduced by a high concentration of GTEE. These mechanistic data indicate that GTEE inhibits the PI3K/Akt and MAPK/ERK signaling pathways associated with cell growth of mPCa.

### 2.4. GTEE Induces Caspase-Dependent Apoptosis in mPCa Cells

**Figure 4:**
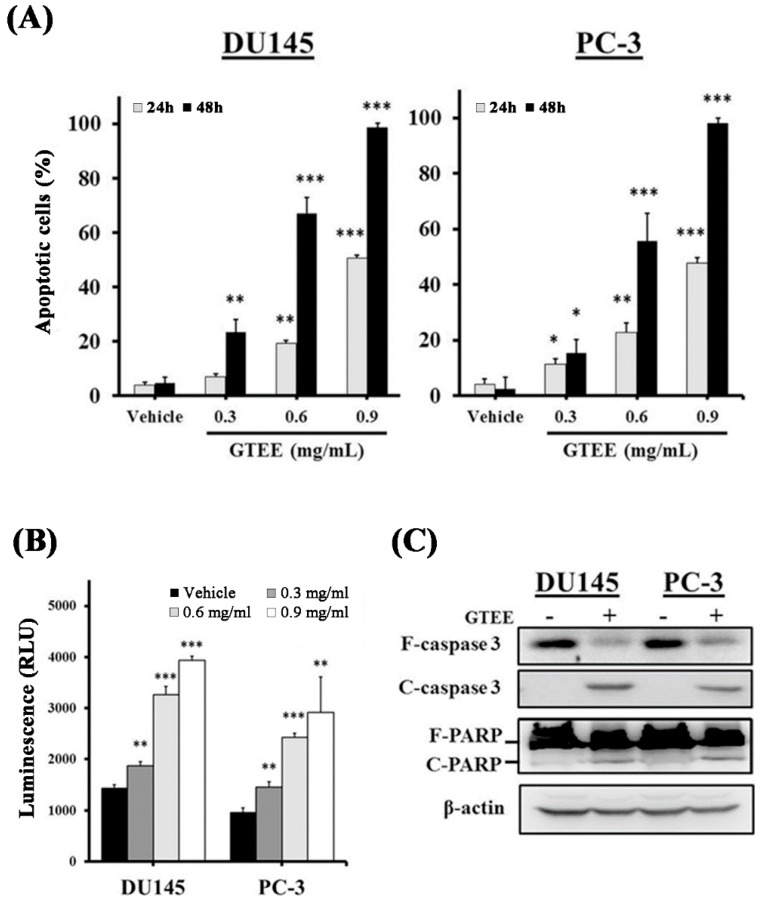
GTEE induces caspase-dependent apoptosis in mPCa cells. (**A**) After 24 or 48 h treatment of vehicle or GTEE (0.3, 0.6 and 0.9 mg/mL), apoptotic cells (%) of DU145 and PC-3 were determined by flow cytometry-based Annexin V-FITC and PI staining analysis. Data are shown as the mean ± SD of three independent experiments. * *p* < 0.05, ** *p* < 0.01, *** *p* < 0.001. (**B**) GTEE induced caspase-3/7 activity in both DU145 and PC-3 cells in a concentration-dependent pattern. Luminescence detection (RLU) of caspase-3/7 activity was measured by an enzymatic activity assay. Results represent the mean ± SD of triplicate experiments. ** *P* < 0.01, *** *P* < 0.001. (**C**) Western blot analysis of apoptosis-related markers (caspase-3 and PARP) in DU145 and PC-3 cells treated with vehicle or GTEE (0.9 mg/mL) for 18 h. GTEE decreased full length (F)-caspase-3 and -PARP, and increased cleaved (C)-caspase-3 and -PARP expression in both mPCa cells. β-actin was used as a loading control.

The inhibition of cell growth can be achieved by inducing cell cycle arrest and/or activating cellular apoptosis [25,26]. Additionally, blockade of the MAPK/ERK pathway leading to activation of caspase-dependent apoptosis in PCa cells has been shown [24]. To further examine if GTEE induces apoptosis through the caspase-dependent pathway in mPCa cells, flow cytometry-based Annexin V-FITC/PI double staining analysis, caspase enzymatic activity assay and Western blotting of caspase expression were conducted. The results of Annexin V-FITC/PI double staining analysis demonstrated that GTEE significantly elevated the percentages of apoptotic cells with a dose-dependent pattern in DU145 and PC-3 cells after 24 or 48 h treatment (Figure 4A). Subsequently, the caspase-3/7 enzymatic activity and expression of caspase-3 and PARP were examined to investigate whether GTEE induced the caspase-dependent apoptotic pathway in PCa cells. As shown in Figure 4B, GTEE increased caspase-3/7 activity in both DU145 and PC-3 cells in a dose-dependent manner (0.3, 0.6 and 0.9 mg/mL) at 8 h treatment. Similar results were observed in Western blot analysis, where GTEE decreased expression of full length (F)-caspase-3 and -PARP as well as increased expression of cleaved (C)-caspase 3 and -PARP in DU145 and PC-3 cells (Figure 4C). These data demonstrate that GTEE induces caspase-dependent apoptotic death in mPCa cells.

### 2.5. GTEE Suppresses PC-3 Tumor Growth in a Subcutaneous Xenograft Mouse Model

**Figure 5:**
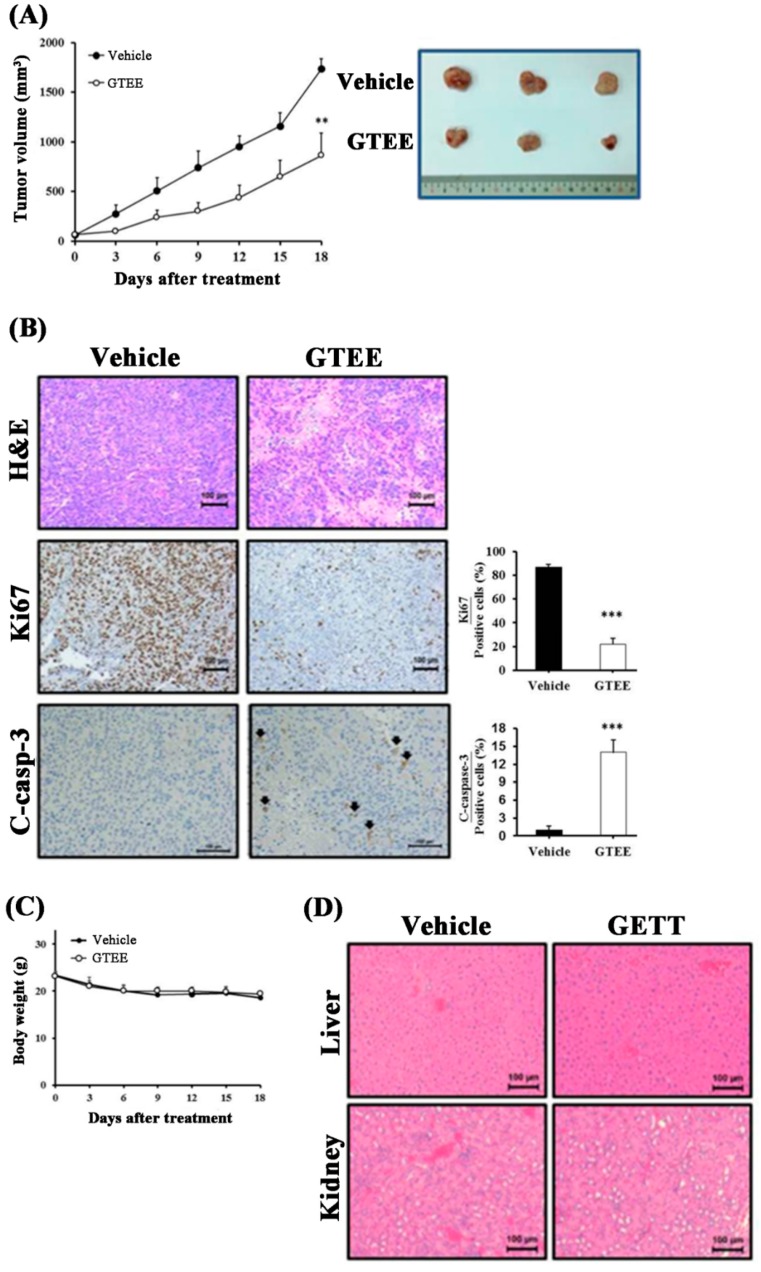
GTEE inhibits PC-3 tumor growth in a subcutaneous xenograft mouse model. (**A**) GTEE significantly inhibits the growth of subcutaneous PC-3 tumors compared to the control of vehicle-treated tumors for 18-day treatment. The tumor volumes (mm^3^) represent as the mean ± SEM (*N* = 5/group). ** *p* < 0.01, significant differences from the vehicle group. A Photograph of representative tumors in each group (Day 18) was shown (right bottom panel). (**B**) IHC staining results demonstrated that significant decrease of Ki67 (cell proliferation) and increase of cleaved (**C**)-caspase-3 (apoptosis) were observed in the GTEE-treated PC-3 tumors compared to the control tumors. Scale bar = 100 µm. Quantifications of Ki67 and C-caspase-3 were determined by counting positive stained cells in an average of five random fields. *** *p* < 0.001. (**C**) Body weights of the vehicle- and GTEE-treated mice during 18-day treatment were recorded. (**D**) H&E staining of liver and kidney harvested from the vehicle- and GTEE-treated PC-3 tumor-bearing mice. No obvious histopathological differences were observed between these two groups in these organs. Scale bar = 100 µm.

Because the in vitro data demonstrated the anti-growth role of GTEE in mPCa cells, we subsequently evaluated the therapeutic potential of GTEE in vivo. A subcutaneous xenograft mouse model of PC-3 tumor growth in male nude mice was established. After the volume of PC-3 tumors reached approximately 100 mm^3^, the tumor-bearing mice were orally administered either GTEE or vehicle control. As shown in Figure 5A, the mice treated with GTEE displayed a significant inhibition in the growth of PC-3-implanted tumors compared to the vehicle control group. To further investigate the underlying molecular basis by which GTEE suppressed PC-3 tumor growth, we determined cell proliferation (Ki67) and apoptosis [cleaved (C)-caspase-3] in tumor tissue sections harvested from the GTEE-treated and vehicle control mice. Significantly decreased expression of a proliferation marker Ki67 and increased expression of an apoptotic marker C-caspase-3 in the GTEE-treated PC-3 tumors compared to the vehicle-treated tumors were observed by IHC staining (Figure 5B).

Bodyweight was monitored to evaluate the general cytotoxicity of GTEE in our xenograft mouse model. As shown in Figure 5C (left panel), there was no significant alteration in the body weights of the mice treated with or without GTEE. Additionally, histopathological results of the liver and kidney sections collected from the GTEE and vehicle control mice showed no signs of toxicity (Figure 5D, right panel). These data indicate that GTEE displayed no obvious cytotoxicity in mice bearing with PC-3 tumors. Collectively, the preclinical results suggest that GTEE effectively inhibited PC-3 tumor growth and could be applied as a potential and safe anti-mPCa agent.

## 3. Discussion

PCa metastatic progression is one of the most significant and unresolved clinical issues in PCa research. Currently, there is no effective therapy to cure this disease. Several traditional Chinese herbal medicines have shown to be promising therapeutic agents for cancer treatment [8,9,13,14,15]. Among these Chinese herbal medicines, *Ganoderma* is the most widely utilized herbal medicine with minimal adverse effects [8]. One of the main species planted in Taiwan, GT, has been demonstrated to display antiproliferative effects on human cancer cells in our laboratory [12,13,14,15]. In the present study, we revealed for the first time that GTEE showed the very promising anti-cancer efficacy on clinically relevant metastatic prostate cells, PC-3 and DU145 cells.

Uncontrolled cell division and proliferation are the hallmarks of cancer [27]. The cell cycle is the major event leading to cell division and proliferation. By deregulating this biological process, cancer cells become masters of their own destiny. Therefore, blockade of the cell cycle has been considered as an attractive therapeutic approach to eliminate cancer cells [27,28,29]. The progression of the cell cycle includes several checkpoints that act as surveillance mechanisms [30]. Checkpoints at G_0_/G_1_ and G_2_/M transitions, as well as S phase, are the essential regulatory gates during the cell cycle progression. Additionally, cyclins are the key protein factors to control the progression of the cell cycle [19]. Interestingly, one of the molecular bases of GT in cancer cells is the alteration of the cell cycle progression [12,13,14,15]. Previous research reported that the methanol fraction of GT induced G_2_/M cell cycle arrest in colorectal cancer cells [15] and the ethanol extract of GT increased S phase arrest in doxorubicin-resistant lung adenocarcinoma cells [13]. In this study, we observed that GTEE showed different effects on the distributions (G_0_/G_1_ and G_2_/M transitions as well as S phase) of the cell cycle in DU145 and PC-3 cells, especially at concentrations of 0.6 and 0.9 mg/mL (Figure 2A,B). However, GTEE decreased expression of cell cycle-related proteins, including cyclins A, B1, D1 and E, in both DU145 and PC-3 cells in a dose-dependent manner (Figure 2C). The various effects of GTEE on the cell cycle could be due to the specificity of different cell types, such as PC-3 cells derived from bone metastasis [17] and DU145 isolated from brain metastasis [18]. Currently, we are attempting to isolate and purify the anti-cancer factors from GTEE. Once the active ingredients are identified, these new compounds in GTEE will be further examined in mPCa cells and other cancer cell lines as potential cell cycle inhibitors to prevent and/or cure deadly cancer aggressiveness in the near future.

Apart from cell cycle arrest, GTEE also showed the induction of programmed cell death in mPCa cells. Apoptosis is a biological process of programmed cell death that occurs in embryonic development, normal cell turnover and cancer cell treatment. Several potential anti-cancer agents have been demonstrated to exert their efficacy on the activation of apoptosis in cancer cells [13,31,32]. Caspase-3 is a main factor that converges the intrinsic (mitochondrial-mediated) and extrinsic (death receptor-mediated) apoptotic pathways in cells [33]. Upon activation of caspase-3, substrates such as PARP are cleaved, ultimately leading to apoptotic cell death. Our data demonstrated dose-dependent induction of apoptosis through activation of caspase-3/7 enzymatic activity in both PC-3 and DU145 cells treated with GTEE (Figure 4B). In addition, Western blotting results showed that GTEE increased expression of C-caspase 3 and C-PARP in PC-3 and DU145 cells (Figure 4C). The activation of these apoptosis-related proteins by GTEE is responsible for the concomitant execution phase of programmed cell death in mPCa cells. Collectively, these findings suggest that checkpoints for cell cycle arrest and apoptosis are targeted by GTEE in mPCa cells. Therefore, GTEE could be applied as a potential therapeutic approach for the treatment of mPCa.

## 4. Materials and Methods

### 4.1. Preparation of Ganoderma Tsugae Extract

*Ganoderma tsugae* (GT) was provided by the Luo-Gui-Ying Fungi Agriculture Farm (with a registered name of Tien-Shen Lingzhi), Taoyuan, Taiwan. Briefly, the powder of the GT fruiting body (30 g) was soaked in 99.9% ethanol (3000 mL), mixed, and shaken for 24 h on a rotating shaker at room temperature under dark. After centrifugation, the supernatant was filtered through Whatman No.1 filter paper (Cat. No. 1001-110), and the residues were extracted with ethanol for an additional two times as mentioned above. The filtrates were collected together and subjected to concentration under reduced pressure (i.e., evaporated to dryness under reduced pressure) to produce a brown gel-like GT extract (GTEE). The yield was approximately 11%. GTEE was then prepared as a stock solution with ethanol solvent (200 mg/mL) and stored at −80 °C until use. Additionally, the quality control of GTEE had been assessed and validated using both chemical fingerprint (HPLC and ESI-MS) [13] and bioresponse fingerprint (PhytomicsQC platform) analyses, as described previously.

### 4.2. Cell Lines and Cell Culture

Human mPCa cell lines PC-3 [17] and DU145 [18] were obtained from the Bioresource Collection and Research Center (BCRC, Hsinchu, Taiwan) and American Type Culture Collection (ATCC, Manassas, VA, USA). All cells were cultured in DMEM/F12 (GIBCO, Paisley, Scotland, UK) medium supplemented with 10% fetal bovine serum (GE, South Logan, UT, USA) at 37 °C in a humidified atmosphere containing 5% CO_2_.

### 4.3. Cell Proliferation and Progression Assays

To assess the GTEE-mediated alteration of prostate cancer cell proliferation and metastatic progression, cell viability assay [13,34], wound healing analysis [13] and cell migration and invasion assays [31,35] were performed as previously described.

### 4.4. Flow Cytometric Analysis

The cell cycle is the key to cell proliferation. To understand the GTEE-mediated effect on the cell cycle, the phase distribution in mPCa cells was detected by flow cytometry as described previously [13]. The mPCa cells were incubated with GTEE or vehicle control for various time points and subsequently fixed with ice-cold 70% ethanol overnight at 4 °C. The cells were washed twice with PBS and incubated with a propidium iodide (PI) solution [13]. The DNA content was measured by flow cytometry (BD FACS Canto, San Jose, CA, USA). The results were analyzed using the FCS Express v2.0 software. Regarding the GTEE-mediated induction of apoptosis in mPCa cells, mPCa cells were stained using an Annexin V-FITC Apoptosis Detection Kit I (BD Biosciences, San Diego, CA, USA) according to the manufacturer’s instruction. The amounts of apoptotic cells were determined by flow cytometry and analyzed by the FACS Express v2.0 software.

### 4.5. Western Blot Analysis

To understand the GTEE-mediated effect on the level of expression of relevant proteins in mPCa cells, the western blotting technique was used. Briefly, the mPCa cells treated with GTEE or vehicle were lysed in lysis buffer (20 mM Hepes buffer pH 7.0, 10 mM KCl, 2 mM MgCl_2_, 0.5% NP-40 and protease inhibitors). Following cell lysis, the supernatants were collected after centrifuging the cell lysates. The total protein concentrations of the supernatant were measured using a Bio-Rad protein assay kit (Bio-Rad Inc., Hercules, CA, USA). Then, 40 µg of total protein extract was prepared and used for polyacrylamide gel electrophoresis. After electrophoresis, proteins on the gel were blotted and transferred onto PVDF membranes. The protein-containing membranes were blocked using 5% nonfat milk in Tris-buffered saline with Tween-20 (TBST) for 1 h at room temperature. After blocking, the PVDF membranes were incubated with primary antibodies for 1 h at room temperature. Before incubation with an HRP-conjugated secondary antibody, the membranes were washed with TBST for 3 times. The reactive signals were visualized using an Enhanced Chemiluminescence Kit (Amersham Biosciences, Arlington Heights, IL, USA). The reactive bands were scanned and quantified using the ImageJ software. Primary antibodies used in this study included anti-Akt (Cell Signaling Technology, Danvers, MA, USA), anti-p-Akt (Ser473; Cell Signaling Technology, Danvers, MA, USA), anti-ERK (Santa Cruz, Dallas, TX, USA), anti-p-ERK (Thr202/Tyr204; Santa Cruz, Dallas, TX, USA), anti-cyclin A, B1, D and E (Santa Cruz, Dallas, TX, USA), anti-caspase 3 (Novus Biologicals, Littleton, CO, USA), anti-cleaved caspase 3 (BioVision, Milpitas, CA, USA), anti-PARP (GeneTex, Irvine, CA, USA) and anti-β-actin (Millipore, Burlington, MA, USA).

### 4.6. Animal Experiments

All animal experiments were performed in accordance with the protocol approved by the China Medical University Institution Animal Care and Use Committee (IACUC) (protocol No. 2017-161-1). Athymic nu/nu male mice (4-week-old) were implanted subcutaneously with PC-3 cells (7.5 × 10^5^ cells). Mice bearing PC-3 tumors with approximately 50–100 mm^3^ in volume were randomly divided into vehicle control (*N* = 5) or GTEE (*N* = 5; 600 mg/kg/body weight) groups with daily feeding by oral gavage for 18 days. The tumor volume and body weight were monitored every 3 days. At the end of the animal experiments (after 18-day treatment), PC-3 tumors were completely excised from the subcutaneous tissue and weighed. Immunohistochemical (IHC) staining was performed on PC-3 tumors for the analyses of Ki67 (cell proliferation) and cleaved caspase-3 (apoptosis) [32,35]. In addition, biochemical and hematological parameters were used to evaluate potential drug toxicity.

### 4.7. Statistical Analysis

All data were analyzed at least three individual experiments by using two-tailed unpaired Student’s *t* test for comparison of independent means. The statistical software was used SigmaPlot (version 11). *P* values of less than 0.05 were considered to be statistically significant.

## 5. Conclusions

In summary, this is an innovative study that GTEE inhibits growth and metastatic capability of mPCa cells in vitro and PC-3 tumor growth in vivo through induction of cell cycle arrest and apoptosis. The data support the possibility that GTEE could be developed as a potentially effective pharmacologic strategy for the treatment of lethal mPCa.

## Abbreviations

ADT	androgen deprivation therapy
FITC	fluorescein isothiocyanate
GTEE	Ganoderma tsugae ethanol extract
mPCa	metastatic prostate cancer
PCa	prostate cancer
TCM	traditional Chinese medicine
